# Zebrafish Models of Parkinson’s Disease: From Pathogenesis to Drug Discovery

**DOI:** 10.3390/ijms27104578

**Published:** 2026-05-20

**Authors:** Hae-Chul Park, Yongbo Seo, Yeo Jeong Han, Su Hee Cho, Myung Ji Kim

**Affiliations:** 1 Department of Biomedical Sciences, Korea University College of Medicine, Seoul 04763, Republic of Korea; hcpark67@korea.ac.kr (H.-C.P.); ybs4428@korea.ac.kr (Y.S.); hst000210@korea.ac.kr (Y.J.H.); 2 Department of Neurosurgery, Korea University College of Medicine, Korea University Medical Center, Ansan Hospital, Ansan 15355, Republic of Korea; shsh36@naver.com

**Keywords:** animal model, α-synuclein, dopaminergic neuron, drug screening, high-throughput screening, neurodegeneration, Parkinson’s disease, zebrafish (*Danio rerio*)

## Abstract

Parkinson’s disease (PD) is a neurodegenerative disorder characterized by the aggregation of Lewy bodies, composed of the protein α-synuclein, and the degeneration of dopaminergic (DA) neurons in the substantia nigra pars compacta. The management of PD seeks to mitigate motor symptoms by substituting diminished endogenous dopamine; nevertheless, it does not halt disease progression. Various animal models have been employed to elucidate the etiology of PD and to discover disease-modifying treatments. Zebrafish serve as a PD model owing to their capacity for high-throughput screening. This review presents updates on the currently available zebrafish models of PD, encompassing both chemically induced and genetically based models, and discusses their advantages and limitations. This review also delineates numerous investigative strategies that utilize the zebrafish PD model and summarizes the findings of previous studies. Taken together, further studies, including the investigation of the regeneration mechanism of DA neurons, neurobehavioral testing of adult zebrafish reflecting PD-associated neurocognitive impairment, and a reliable gene-based model providing precise gene knockout and reproducibility, may assist in elucidating the critical pathways that trigger PD and its progression, alongside potential targets to hinder this progression.

## 1. Introduction

Parkinson’s disease (PD) is characterized not only by α-synuclein (αSyn) aggregation and dopaminergic (DA) neuronal degeneration, but also by multiple interconnected pathogenic mechanisms, including mitochondrial dysfunction, oxidative stress, neuroinflammation, impaired autophagy–lysosomal degradation, calcium imbalance, and disrupted protein homeostasis [1,2,3,4,5,6]. In particular, mitochondrial impairment and oxidative stress have been strongly implicated in DA neuronal vulnerability [5]. In contrast, defects in autophagy and lysosomal degradation contribute to impaired αSyn clearance and accumulation of misfolded αSyn [3]. In addition, accumulating evidence suggests that αSyn pathology may propagate across interconnected neuronal networks in a prion-like manner, thereby contributing to disease progression [7,8,9]. Neuroinflammatory responses mediated by activated microglia and astrocytes have also been recognized as important contributors to PD pathogenesis [2]. Furthermore, disruption of mitochondrial calcium homeostasis and mitophagy-associated pathways involving PINK1, Parkin, DJ-1, and LRRK2 has been associated with neuronal degeneration in familial and sporadic forms of PD [4,10,11,12,13,14]. Patients with PD exhibit a combination of motor (bradykinesia, rigidity, resting tremor, postural instability, and gait disturbance) and non-motor (constipation, hyposmia, depression, cognitive decline, and sleep disturbance) features that significantly affect their quality of life and caregiver requirements [15]. These symptoms become more severe as the disease progresses and are accompanied by exacerbation of dopamine deficiency. The current aim of PD treatment is to alleviate motor symptoms by substituting the reduced endogenous dopamine with levodopa and other DA modulators. Nonetheless, with time, the drug’s efficacy diminishes (“wearing off”), and adverse side effects intensify with increased dosage and frequency of administration. Although there is a need for disease-modifying treatments that alter the progression of PD, none are currently available. While research on the etiology of PD should be conducted prior to the development of disease-modifying treatments, the etiology and progression of PD are known to be influenced by environmental and genetic factors [1]. Nevertheless, the precise mechanism underlying PD pathophysiology remains unresolved and is currently being investigated. Compared with several other neurodegenerative disorders, PD is particularly well suited for zebrafish-based modeling because many cardinal pathological and behavioral features of PD can be effectively reproduced and quantitatively analyzed in zebrafish [16,17,18,19]. Importantly, zebrafish possess evolutionarily conserved DA pathways and neurotransmitter systems that share substantial anatomical, molecular, and functional similarities with those of humans, thereby enabling effective investigation of DA neuronal degeneration and dopamine-associated behavioral abnormalities [20,21,22]. As highlighted by previous studies, zebrafish have increasingly emerged as valuable vertebrate models for PD research owing to their conserved DA pathways, rapid embryonic development, optical transparency, amenability to genetic manipulation, and suitability for high-throughput phenotypic drug screening [18,23,24,25,26]. In addition to summarizing chemical and genetic zebrafish PD models, this review provides a comprehensive overview of the investigative methodologies used to evaluate these models, including locomotor-behavioral assays, imaging techniques, gene expression analyses, Western blot, and proteomic approaches. By integrating experimental strategies with representative findings, this review aims to provide a practical framework that may guide researchers in designing and optimizing zebrafish PD models and related experimental studies for mechanistic investigation and disease-modifying drug discovery.

## 2. The Zebrafish Model for the Drug Screening of Parkinson’s Disease

Zebrafish embryos possess transparency and facilitate real-time in vivo monitoring of neuronal injury in living specimens using neuron-specific fluorescent reporter lines. In addition, owing to their rapid reproduction, zebrafish embryos and larvae are ideal for high-throughput phenotyping and large-scale in vivo study [23,26]. Moreover, neurotoxins can be readily delivered via immersion, facilitating straightforward and consistent exposure regimens [18]. The embryonic and larval phases of zebrafish have distinct benefits in mimicking the initial neurodegenerative processes. During this developmental period, the nervous system grows rapidly, and the DA circuitry fully functions and reacts to neuroactive substances that function in the central nervous system (CNS) [20,27]. However, neurotoxin exposure during early developmental stages may cause systemic developmental abnormalities in addition to DA neurotoxicity [28,29]. They still serve as important animal models for investigating PD processes and developing novel pharmaceuticals. In contrast, adult zebrafish possess mature neural circuits and more stable DA systems, enabling evaluation of complex behavioral phenotypes and neurodegenerative pathology following neurotoxin administration [30]. Adult zebrafish models may therefore provide greater translational relevance to late-onset PD phenotypes [31]. In addition, adult zebrafish allow the use of administration methods such as intraperitoneal or intracranial injection, which may improve targeting specificity [32]. However, adult neurotoxin models are generally more labor-intensive, lower in throughput, and may require technically demanding procedures compared to embryonic and larval models [32,33]. Regarding genetic models, the majority of prior research on neurodegenerative diseases utilizing zebrafish models employed transitory knockdown (KD) approaches of pathogenic genes through the injection of morpholino oligonucleotides (MO) into embryos. MO-based transient KD approaches may produce variable phenotypes due to transient gene suppression, off-target effects, and differences in experimental conditions [34,35]. Consequently, to effectively investigate the pathophysiology of PD using zebrafish models, stable transgenic and knockout (KO) models using methods such as clustered regularly interspaced short palindromic repeats-associated protein 9 (CRISPR/Cas9) that generate targeted genetic modifications of genes implicated in the disease are important [36,37,38]. Therefore, it is crucial to create a reliable zebrafish model of PD. Zebrafish are more amenable to genetic manipulation than other laboratory animals [18], providing considerable benefits for the development of disease models. Furthermore, owing to the unidentified etiology of neurodegenerative diseases, such as PD, conventional drug screening techniques based on pharmacological target identification have proven ineffective. Consequently, to formulate a disease-modifying treatment for PD, it is essential to establish a precise model of the condition and to perform phenotypic screening to identify potential drug candidates that promote recovery [39]. Although both chemical and genetic zebrafish PD models reproduce important pathological and behavioral features of PD, careful model selection is necessary depending on the experimental objective and targeted disease mechanism. A comparative summary of representative chemical and genetic zebrafish PD models is presented in Table 1. Consequently, zebrafish offer considerable benefits in the development of disease models and the exploration of new pharmaceuticals.

## 3. Zebrafish Parkinson’s Disease Model

### 3.1. Chemical Model

#### 3.1.1. 1-Methyl-4-Phenyl-1,2,3,6-Tetrahydropyridine (MPTP)

Zebrafish were developed as PD models subjected to several neurotoxins, as listed in Table 2. The 1-Methyl-4-Phenyl-1,2,3,6-Tetrahydropyridine (MPTP)-induced PD zebrafish model has been extensively utilized to screen anti-PD drugs and to explore their underlying pathophysiology [40,41,42]. Neurotoxic MPTP selectively induces degeneration of DA neurons in the substantia nigra by blocking mitochondrial complex I activity, resulting in increased oxidative stress and neuronal apoptosis via the metabolite 1-methyl-4-phenylpyridinium (MPP^+^) [28,43,44]. In the zebrafish brain, dopamine transporter (DAT)-expressing neurons selectively capture MPP^+^, which accumulates in the DA cell bodies of the ventral diencephalon (vDC) [20]. This results in the degradation of DA neurons, ultimately leading to apoptosis and a significant decrease in striatal dopamine levels. Zebrafish larvae exhibit DA neurodegeneration in response to both MPTP and MPP^+^; however, MPTP results in more significant locomotor impairments and morphological anomalies, likely attributable to systemic exposure and secondary metabolites [28], while MPP^+^ leads to similar neuronal loss with comparatively less severe behavioral impairments [29]. MPTP is commonly utilized to replicate PD in zebrafish via aqueous administration because its lipophilic nature enables efficient penetration of the blood–brain barrier (BBB) [31,45]. Given that the BBB in zebrafish is structurally and functionally analogous to that in higher vertebrates, and that it develops by 3 days post-fertilization (dpf), it is imperative to ascertain whether chemicals are able to cross the BBB after 3 dpf when establishing a zebrafish PD model using a chemical toxin or conducting drug screening with this model [23,46,47]. Using an adult zebrafish model of PD, MPTP has been injected intraperitoneally [48,49,50], intramuscularly [51], and intraventricularly [33]. A recent study by Ye et al. presented a MPTP-induced zebrafish PD model exhibiting reduced number of DA neurons, reduced motility, decreased neuronal vasculature, downregulation of mitophagy-related gene expression, upregulation of peroxidation-related gene expression, and downregulation of antioxidant gene expression, which were reversed by co-administration with Epicoccin A [52]. Recently, Maleski et al. demonstrated the molecular characterization of MPP^+^-induced neurotoxicity in zebrafish larvae via label-free quantitative proteomics [53]. Proteomic analysis by the researchers. demonstrated increased proteasome components and antioxidant proteins as early stress responses, along with downregulation of mitochondrial enzymes and synaptic regulators, reflecting characteristic neurodegenerative processes associated with PD [53]. Recent findings from Intonti et al. [54] showed that intraperitoneal administration of MPTP in adult zebrafish triggered a temporary infiltration of CD4^+^ T cells, accompanied by astroglial reactivity and microglial activation. Furthermore, immunofluorescence-based analyses demonstrated an inverse relationship between CD4^+^ T-cell abundance and TH^+^ DA neuron levels [54], indicating that immune responses may contribute to both degenerative and regenerative events in the zebrafish PD model [55,56].

#### 3.1.2. 6-Hydroxydopamine (6-OHDA)

6-Hydroxydopamine (6-OHDA) is a hydroxylated compound of dopamine that is taken up by DAT into DA neurons, inducing mitochondrial dysfunction, reactive oxygen species (ROS) production, and ultimately resulting in neurodegeneration [24,25]. Upon entering neurons, monoamine oxidase (MAO) oxidizes 6-OHDA to a quinone along with byproducts such as hydrogen peroxide and various free radical compounds [24,57]. Dopamine oxidation induces mitochondrial and lysosomal dysfunction, partly by inhibiting the activity of glucocerebrosidase, a lysosomal enzyme associated with PD pathogenesis, and by increasing mitochondrial hydrogen peroxide levels [58,59]. Due to its inability to traverse the BBB and access the brain without direct injection, 6-OHDA is less frequently employed than MPTP in neuroscience research [51,60]. To examine the localized effects on DA neurons, 6-OHDA is often administered directly to various brain areas [24]. A recent study injected 6-OHDA into the dorsal telencephalic ventricle of adult zebrafish, specifically targeting DA periglomerular neurons in the olfactory bulb, to establish a model of early PD-related olfactory dysfunction without locomotor deficits, thereby recapitulating the characteristics of the prodromal stage of the disease [61]. The authors indicated that olfactory impairment and synaptic degeneration were restored by 7 days post-injection (dpi), emphasizing the distinctive regenerative capacity of zebrafish [61]. Regarding the regenerative capabilities of zebrafish, another study using adult male zebrafish lesioned by microinjecting 6-OHDA into the ventral vDC also demonstrated the full recovery of DA neurons 30 days post-lesion [60]. Chemically induced zebrafish models are inherently transient, as prolonged toxin exposure can increase mortality [30,48], whereas intermittent or acute exposure may trigger endogenous regenerative responses in the zebrafish brain [62,63]. However, the regenerative capabilities of zebrafish may hamper the establishment of long-term PD models. In contrast, the remarkable regenerative capabilities of zebrafish can be utilized to develop disease-modifying treatments for PD. This provides a compelling basis for elucidating regenerative mechanisms and potentially applying them to non-regenerative mammals such as humans [62]. For instance, studies on axon regeneration in the CNS of zebrafish have demonstrated significant intrinsic axon growth potential, limited scar formation, reduced expression of growth inhibitors, and guidance of regenerated axons by molecular signals [64]. Kyritsis et al. proposed that acute inflammation facilitates CNS regeneration by supplying essential signals for the onset of reactive proliferation and regenerative neurogenesis in the adult zebrafish brain, subsequent to the identification of the signaling pathway in zebrafish that links the inflammatory response to the effective augmentation of stem cell activity and commencement of neural regeneration [63]. We anticipate that zebrafish will aid in clarifying the molecular mechanisms governing CNS regeneration, resulting in functional reconnections within the vertebrate CNS.

#### 3.1.3. Rotenone

The pathogenesis of PD is believed to involve mitochondrial respiratory chain malfunction, which specifically affects complex I and ROS generation. Rotenone directly inhibits mitochondrial complex I [65]. A previous study indicated that PD occurred 2.5 times more frequently in individuals who reported rotenone use than in non-users [66]. Despite the belief that rotenone has a relatively short environmental half-life and restricted bioavailability [67,68], persistent exposure to rotenone in laboratory settings has been documented to exert additional effects associated with the etiology of PD [69,70]. Exposure to rotenone effectively elicits PD-like symptoms in zebrafish, including diminished locomotor activity, reduced dopamine levels, elevated oxidative stress, intensified inflammatory response, decreased population of DA neurons, and compromised mitochondrial function [16,71,72,73,74]. Mitochondrial Ca^2+^ levels are associated with PD etiology [4]. The degradation of calcium homeostasis is attributed to the activation of mitochondrial permeability pores due to calcium influx into the mitochondrial matrix, which is contingent on the membrane potential [75,76]. Sig-1R, a chaperone protein located in the mitochondria-associated endoplasmic reticulum membrane that facilitates calcium signaling between two organelles, is essential for modulating Ca^2+^ levels in the ER-mitochondria-associated membrane, sustaining intracellular Ca^2+^ homeostasis, and facilitating Ca^2+^ uptake into the mitochondrial matrix [77,78]. A previous study using a rotenone-induced zebrafish model of PD reported a significant reduction in mitochondrial calcium levels in the rotenone group, with a significant compensatory increase in *Sig-1R* expression [79]. Using an adult zebrafish rotenone-induced PD model, Parthiban et al. demonstrated through hematoxylin and eosin histological examination that rotenone treatment caused prominent neurodegenerative alterations, including neuronal loss, cytoplasmic vacuolar changes, necrotic features, and disruption of normal brain tissue architecture [80].

#### 3.1.4. Paraquat

Epidemiological research has indicated that paraquat (PQ) increases the likelihood of acquiring PD [81,82,83,84,85]. PQ is a widely established inducer of oxidative stress, and oxidative stress resulting from the redox cycling of PQ is generally considered the primary contributor to its toxicity [86,87]. A previous study describing the mechanism by which PQ^2+^ enters DA neurons reported that PQ^2+^, in its original divalent cation form, does not serve as a substrate for DAT [88]. However, upon conversion to the monovalent cation PQ^+^ by a reducing agent or NADPH oxidase in microglia, it becomes a substrate for DAT and is sequestered in DA neurons, where it elicits oxidative stress and cytotoxicity [89]. Significant oxidative stress and antioxidant gene expression were activated following exposure to a median lethal concentration (LC_50_) [90,91]. Additionally, apoptosis, specific macrophage activation, and migration were observed for the first time in the LC_50_ PQ-exposed zebrafish embryos and larvae [90]. Nellore et al. subjected zebrafish embryos to PQ, which resulted in motor impairments at the larval stage as well as reductions in dopamine and serotonin; elevations in malondialdehyde (MDA), a marker of lipid peroxidation; a decrease in glutathione (GSH); and an increase in apoptotic cells stained with acridine orange [92]. Bortolotto et al. found that adult zebrafish administered an intraperitoneal injection (i.p.) of either 10 mg/kg (Pq10) or 20 mg/kg (Pq20) PQ every 3 days for a total of six injections exhibited significant changes in swimming behavior compared to that in controls; however, no abnormalities in spatial memory, anxiety, or social interaction were observed [30]. Conversely, an alternative study indicated the presence of anxiolytic and aggressive behaviors; however, no motor deficits were noted in i.p. Pq20 injected adult zebrafish [91].

### 3.2. Genetic Model

#### 3.2.1. Alpha-Synuclein (SNCA)

The abnormal accumulation of LB (Lewy body), a proteinaceous inclusion composed of fibrillary aggregates of αSyn, is a characteristic neuropathological feature of PD [93]. Substantial evidence indicates that the accumulation of αSyn pathogenic aggregates is causally linked to the emergence of motor and non-motor symptoms in PD [94,95]. Previous studies have demonstrated that αSyn has a regulatory role in synaptic signaling [96]. Research in animal models of PD has demonstrated that αSyn deposition can damage neurons and ultimately cause neuronal apoptosis by causing significant synaptic impairments, disrupted mitochondrial homeostasis, lysosomal dysfunction, disrupted membrane, and endoplasmic reticulum stress [3,97,98,99]. Understanding the molecular mechanisms that regulate LB formation and αSyn toxicity is crucial for elucidating the pathophysiology of PD [93,98]. The primary pathogenic role of αSyn in PD is supported by genetic research indicating that mutations and duplications in the αSyn gene locus, *SNCA*, can trigger the onset of autosomal-dominant familial variants of the disease [100]. Despite *SNCA* being a highly conserved gene, the zebrafish is a vertebrate that noticeably lacks it. Instead, three zebrafish genes—*sncb*, *sncga*, and *sncgb* (which encode β-, γ1-, and γ2-synucleins, respectively)—have significant evolutionary conservation in relation to their human paralogues [101]. Zini et al. present a stable transgenic line expressing human wild-type αSyn with an N-terminal mCherry tag, expressing the pathologic features of PD, including αSyn deposition, tyrosine hydroxylase (TH)-positive neuron degeneration, alterations of autophagy markers, synaptic protein alterations, motility deficits, and anxiety phenotype in the larvae stage at 5dpf [102]. Point mutations leading to the overexpression of the αSyn gene encompass A30P and A53T [103]. Lopez et al. [104] presented a zebrafish transgenic line expressing wild-type and A53T αSyn with the Dendra2 fluorescent reporter via the GAL4 system. Although these models partially recapitulated PD-related phenotypes, including fibrillary αSyn formation and neuronal death, selective dopaminergic neurodegeneration and PD-associated behavioral abnormalities were not comprehensively characterized. To establish stable αSyn transgenic zebrafish models, it is optimal to seek a balance between a phenotype that is excessively severe and lethal and one that is insufficient to elicit PD symptoms. It is essential to adequately exhibit the early phenotype, specifically neurodegeneration, in larvae when performing drug screening for PD-modifying treatments. This can be efficiently achieved by employing a robust transactivator system.

Our laboratory is currently developing a zebrafish model of PD based on αSyn overexpression using a transcriptional activation system in which the QF transcription factor interacts with the QUAS promoter to enhance target gene expression [105].

#### 3.2.2. Parkin (PD Protein 2 Gene, PARK2)

The genetic basis for the etiology of PD seems especially significant in early-onset cases [106]. *Parkin* is thought to be responsible for the pathogenesis of autosomal recessive juvenile Parkinsonism (AR-JP) [10]. Parkin is an E3 ubiquitin ligase, essential for mitophagy and implicated in the ubiquitination pathway of misfolded proteins originating from the endoplasmic reticulum, thus contributing to protection against neurotoxicity caused by unfolded protein stress [107]. Parkin ubiquitinates the αSyn-interacting protein synphilin-1, and co-expression of parkin, synphilin-1, and αSyn leads to the formation of ubiquitin-positive cytosolic inclusions resembling LBs [108]. Parkin modulates mitophagy, which degrades mitochondria impaired by the PINK1/Parkin pathway, cellular viability, and immunological responses, all of which are disrupted in PD [109]. Furthermore, parkin may influence the dissemination and proliferation of αSyn by regulating its post-translational modifications [110]. Wilkaniec et al. proposed that αSyn oligomers influence the expression, post-translational modification, and functionality of parkin [111]. Subsequent investigations by the same authors indicated a direct correlation between Syn-mediated parkin depletion and compromised mitochondrial activity [112]. In a condition characterized by the accumulation of αSyn, enhancing parkin function may represent an innovative therapeutic approach to avert mitochondrial dysfunction and the progression of PD [110]. The zebrafish *parkin* gene was cloned, revealing that the protein exhibits 62% identity with human parkin and 78% identity with the relevant brain regions [19,113]. Flinn et al. [113] developed an MO-mediated KD of the *parkin* zebrafish model, which selectively affected the number of ascending DA neurons and demonstrated reduced mitochondrial respiratory chain complex I activity and increased susceptibility to MPP^+^, with preservation of swimming ability compared to that of the control. Additionally, the authors observed electron-dense material in the T-tubules of *parkin* KD zebrafish embryos [113]. T-tubules are abundant in the L-type Ca^2+^ channels [114]. Calcium-dependent pacemaking via L-type Cav1.3 calcium channels in nigral neurons is reportedly associated with increased mitochondrial ROS [2,115]. In this context, CaV1.3-selective L-type calcium channel antagonists have been investigated for their neuroprotective effects by diminishing mitochondrial and oxidative stress in DA neurons in PD [76,116]. Clinical trials have been conducted using candidate drugs that modulate calcium homeostasis via this channel [116,117,118,119,120,121]. A potential disease-modifying treatment for PD is reinstating mitophagy and diminishing harmful αSyn accumulation through the modulation of Parkin activity.

#### 3.2.3. PTEN-Induced Putative Kinase 1 (PINK1)

Recessive mutations in *PINK1* (PTEN-induced kinase 1) result in the targeted degradation of DA neurons in the substantia nigra, which is indicative of PD [13,14]. PINK1 is a kinase localized to the mitochondria, and the loss of PINK1 function can modify mitochondrial morphology and dynamics, thereby establishing a connection between mitochondrial dysfunction and the genesis of PD [122,123,124,125,126]. Valente et al. demonstrated that mutations in *PINK1* are linked to *PARK6* (chromosome 1p36), causing a rare form of familial PD, following the identification of two homozygous mutations affecting the PINK1 kinase domain in three consanguineous *PARK6* families [13]. Weihofen et al. [122] demonstrated that Miro, an atypical GTPase, and Milton, an adaptor protein, mitigated altered mitochondrial morphology resulting from PINK1 function loss in cell culture. Their findings indicated that PINK1 was involved in the transport of mitochondria within cells [122]. Gandhi et al. [14] demonstrated that PINK1 modulates calcium efflux from mitochondria through the mitochondrial Na^+^/Ca^2+^ exchanger in PINK1-deficient mammalian neurons. Consequently, the absence of PINK1 leads to mitochondrial calcium accumulation, culminating in calcium overload and increased neuronal susceptibility to cell death [14]. With *pink1*-MO KD in zebrafish, Priyadarshini et al. [127] verified that mild oxidative stress induced by the free radical generator H_2_O_2_ resulted in a significant reduction in *th2* expression, while *th1* expression remained relatively unaffected. Furthermore, L-glutathione was shown to effectively restore the diminished expression of both *th2* and *th1* in *pink1* morphants [127]. Anichtchik et al. observed a general developmental delay and a reduction in the number of neurons in the DA system, alongside elevated glycogen synthase kinase (GSK) 3β activity, a phenotype that was ameliorated by a GSK3β inhibitor [128]. Xi et al. [129] demonstrated that *pink1*-MO zebrafish exhibited diminished responsiveness to tactile stimuli and a significant reduction in swimming distance and velocity. However, no significant differences were observed in the number of DA neurons in the vDC. Additionally, DA neurons in the vDC of *pink1*-MO zebrafish were markedly disorganized, positioned more ventrally, more dispersed, and displayed either shortened axonal projections or a complete absence of projections to the forebrain [129].

#### 3.2.4. DJ-1 (PD Protein 7 Gene, PARK7)

*DJ-1* was initially recognized as an oncogene, with its expression elevated in several cancer types; its oncogenic potential may be associated with the regulation of the phosphatase and tensin homolog (PTEN) tumor suppressor [130,131]. Through homozygosity mapping of two consanguineous families from distinct populations in the Netherlands and Italy, Bonifati et al. identified the *PARK7* gene, which is associated with autosomal recessive early-onset PD and maps to chromosome 1p36 [11,12]. DJ-1 is expressed ubiquitously; in a typical human brain, it is expressed at moderate levels in neurons and astrocytes of the CNS [132]. DJ-1 transitions to a more acidic (oxidized) form in the brains of individuals with PD [132]. Unlike αSyn, DJ-1 does not accumulate in LB but has been detected in Pick bodies [132,133,134]. Bai et al. [135] cloned and characterized the zebrafish ortholog of *dj-1*, demonstrating 83% sequence identity with human *DJ-1*. Additionally, DJ-1 expression was observed in DA neurons within major forebrain and diencephalic TH-positive cell populations in zebrafish. Bretaud et al. [136] demonstrated that MO-injected zebrafish embryos with *dj-1* KD exhibited elevated expression levels of *p53* and *Bax*. Furthermore, concurrent KD of *dj-1* and the negative regulator of p53, mdm2, induced DA neuronal cell death even in the absence of toxin exposure, suggesting that p53 contributes to neuronal cell loss associated with DJ-1 deficiency [136].

A significant discovery was that *dj-1* KD does not affect the number of DA neurons in zebrafish embryos, which is consistent with another study [137]; however, these neurons exhibit heightened sensitivity to oxidative stress and greater vulnerability to proteasome inhibition [136]. A proteomic study of *dj-1* KO zebrafish early adult brains revealed that 5% of the 4091 identified proteins were affected by the absence of DJ-1 [138]. The dysregulated proteins primarily pertain to those associated with mitochondrial metabolism, mitophagy, stress responses, redox regulation, and inflammation [138]. More recently, Solheim et al. established a *park7* KO larval zebrafish PD model that exhibited not only motor deficits, including reduced locomotor activity, but also non-motor abnormalities such as increased sleep latency, elevated daytime sleep ratio, and impaired touch-evoked responses, together with progressive loss of TH positive DA neurons within the DC1 diencephalic region, highlighting the relevance of PARK7-deficient zebrafish for investigating diverse PD-associated neurobehavioral phenotypes [139].

#### 3.2.5. Leucine-Rich Repeat Kinase 2 (LRRK2)

Following the identification of *PARK8* in 2002 by Funayama et al. [140], Ruiz et al. provided persuasive evidence linking mutations in *PARK8*, which encompasses a leucine-rich repeat, kinase domain, RAS domain, and WD40 domain, as the first genetic etiology of late-onset familial and sporadic PD [141]. A missense mutation was identified in the kinase domain of leucine-rich repeat kinase 2 (LRRK2) in individuals with autosomal dominant PD from the Japanese Sagamihara family, which was the foundation for establishing the *PARK8* locus [142]. The kinase activity of LRRK2 may be a crucial factor in the accumulation and aggregation of unfolded proteins in degenerating neurons, particularly in relation to αSyn and tau pathology [143]. Pathologic αSyn stimulates *LRRK2* expression and kinase activity in monocytes, facilitating their recruitment to the brain [144]. αSyn can directly activate microglia and can be cleared by them, whereas LRRK2 is associated with the intrinsic regulation of microglial activation and lysosomal degradation processes [145].

In addition, LRRK2 interacts with various essential regulators of mitochondrial fission and fusion, potentially affecting mitochondrial function directly or indirectly through autophagy or cytoskeletal dynamics [146]. LRRK2 mutations have been reported to increase vulnerability to oxidative stress and apoptosis [147,148]. Pathogenic *LRRK2* mutants exhibit enhanced dendritic and mitochondrial calcium uptake in cortical neurons and fibroblasts from patients with familial PD by upregulating mitochondrial calcium uniporter levels [149]. Co-immunoprecipitation revealed that LRRK2 interacts with parkin. The coexpression of *LRRK2* and *Parkin* augments cytoplasmic protein aggregates containing LRRK2 and intensifies the ubiquitination of these aggregates [150].

Researchers have revealed that MO-injected *lrrk2* KD zebrafish embryos exhibit reduced LRRK2 levels, resulting in a range of developmental anomalies, including several organ abnormalities and loss of DA neurons [151]. *Lrrk2* KD leads to the accompanying up-regulation of β-synuclein, PARK13, and superoxide dismutase 1 (SOD1), resulting in β-synuclein aggregation in the diencephalon, midbrain, hindbrain, and postoptic commissure [151]. Another study demonstrated that MO-injected *lrrk2* KD zebrafish embryos exhibited embryonic mortality and significant developmental anomalies, including growth retardation and neuronal loss [152]. Deletion of the WD40 domain of zebrafish *lrrk2* through MO-mediated splicing did not result in significant embryonic developmental anomalies; instead, it elicited PD-like characteristics, such as degeneration of DA neurons in the diencephalon, disruption of axon tracts, and locomotor impairment [152]. Table 3 summarizes the findings of the literature utilizing genetic-based zebrafish models for PD.

**Table 2:** Chemical-induced Parkinson’s disease zebrafish models.

Neurotoxin	Zebrafish	Dosage/ Administration Period/ Route of Administration	Locomotor- Behavior	Imaging	Additional Results	Reference
MPTP	Larvae	60 µM/ 24–96 hpf/ Immersion	↓ motility (distance, speed)	-↓ length in the subset of DA neurons -Pronounced loss and disorganization of neuronal vasculature -Significant overproduction of ROS	<RT-qPCR> -↑: *αSyn*, *tuba1b*, *syn2α*, peroxidation-related genes—*sod1* and *2* -↓: Mitophagy-related gene—*PINK1*, *Parkin*, *hoxb1a*, *atg7*, *atg12*, *ulk1b*, *beclin1*, *ambra1a*, *lc3b*, antioxidant genes—*gss*, *gsto2*, *gpx4a*, *cat*	[52]
MPP^+^	Larvae	500 µM/ 1–5 dpf/ Immersion	-Hypokinesia -Near-complete absence of light-evoked response		<Proteomics> -Protein-protein mapping: coordinated dysregulation of DJ-1, SDHA, and multiple 26S proteasome subunits -↑: proteasome components and antioxidant proteins (early stage stress response) -↓: mitochondrial enzymes and synaptic regulators	[53]
MPTP vs. MPP^+^	Larvae	MPTP: 0.25 mM (LC_50_) MPP^+^: 0.05 mM (LC_50_)/ 3–7 dpf/ Immersion	-MPTP: ↓ ↓ locomotion -MPP^+^: ↓ locomotion	-MPTP: 39% DA neuron loss in vDC, Strong degeneration in clusters 8 and 13 -MPP^+^: 36% DA neuron loss in vDC	<RT-qPCR> -↑: *p53* -↓: *th1*, *dat* -MPTP: Severe morphological defects (cardiac edema, tail curvature), Strong systemic toxicity -MPP^+^: No major morphological defects, More selective DA toxicity	[29]
6-OHDA	Adult (6–12 months)	10 mM (0.5 µL injection)/ single injection, analyzed at 1–7 dpi/ Intracerebroventricular injection	-Impaired olfactory responses to cadaverine (restored by 7 dpi) -No impairment of motor function	-Loss of DA periglomerular neurons in OB by injecting 6-OHDA into the dorsal telencephalic ventricle -By 7 dpi, remodeling of DA synapses in the OB (SV2 staining) -GFAP+ peaking at 3 dpi: inflammatory response -Significant ↑ Lcp1+ cells: microglial and leukocytic activation -At 1 dpi, significant ↑ TUNEL+ profiles in the OB (by 3 dpi, returned to control level)		[61]
	Larvae	250 µM/ 2–4 dpf/ Immersion	-↓ locomotor activity -↑ time spent in the bottom zone	-↓ TH in whole mount immunostaining	<RT-qPCR> -↑: *TNF-a* and *cd11b* -↓: *parkin* and *pink1* <Western blot> -↑: TNF-a and cd11b -↓: TH	[153]
Rotenone	Adult (4 months)	5 µg/L/ 28 days/ Immersion	-Impairment of locomotor activity -↑anxiety-like behavior	↓ TH^+^ cells across all brain regions	<RT-qPCR> -↑: *th1*, *th2*, proinflammatory cytokines (*il1a*, *il1b*, *tnfa and cox2*) -↓: *dat*, *bdnf* <ELISA> -↓dopamine levels <Mitochondria function assay> -Significant resistance to complex II inhibitors (malonate and carboxin) and meclizine, compared to the control <lipid peroxidation (TBARS production)> -Significant increase in TBARS levels	[71]
	Young adult (6 months)	2 mg/kg/ 3 weeks/ Dissolved in sunflower oil	↓ locomotor activity		<RT-qPCR> -↑: *pink1*, *lrrk2*, *vdac*, *tnfa*, *il21*, *nkap*, *sig-1R* -↓: *tlr4a* <Western blot> -↑: sig-1R -↓: PARK7, γSyn <Mitochondrial calcium levels inductively coupled plasma-optical emission spectrometry (ICP-OES)> -↓ calcium amount in the mitochondria.	[79]
Paraquat	Larvae	0.04 ppm (LC_50_)/ 18–96 hpf/ Immersion	-↓ locomotor activity -↓ spontaneous tail coiling	↑ apoptotic cells (acridine orange staining) with increases in the duration of exposure to paraquat	<Morphology> -Significant neurodegenerative phenotypes like bent tail structure, curved spine structure, and distorted yolk sac <Neurochemical measurement> -Significant ↓ dopamine and serotonin levels <Analysis of lipid peroxidation and glutathione level> -↑MDA ↓GSH	[92]
	Adult (male) (0.4–0.6 g)	10 or 20 mg/kg/ every 3 days × 6 injections/ Intraperitoneal injection	-Alterations in swimming behavior -Changes in the absolute turn angle -No significant difference in anxiety and social interaction -Impaired acquisition and consolidation of spatial memory in the Y-maze task		<RT-qPCR> -↓: *dat* in Pq10-treated group (no significant changes in the Pq20-treated group) <Western blot> No changes in the level of total TH protein <Liquid chromatography-tandem mass spectrometry> -Dopamine ↑ -DOPAC ↓	[30]

↑, increase/upregulation; ↓, decrease/downregulation; MPTP, 1-Methyl-4-Phenyl-1,2,3,6-Tetrahydropyridine; αSyn, α-synuclein; dpf, day post-fertilization; hpf, hour post-fertilization; DA, dopaminergic; ROS, reactive oxygen species; MPP^+^, 1-methyl-4-phenylpyridinium; SDHA, succinate dehydrogenase; 6-OHDA, 6-Hydroxydopamine; dpi, day post-injection; OB, olfactory bulb; vDC, ventral diencephalon; SV2, synaptic vesicle protein 2, GFAP, glial fibrillary acidic protein; Lcp1, lymphocyte cytosolic protein 1; TH, tyrosine hydroxylase; DAT, dopamine transporter; Pq, paraquat; DOPAC, 3,4-dihydroxyphenylacetic acid; GSH, glutathione; MDA, Malondialdehyde; TUNEL, terminal deoxynucleotidyl transferase dUTP nick-end labeling.

**Table 3:** Genetic-based Parkinson’s disease Zebrafish models.

Genes	Zebrafish	Locomotor- Behavior	Imaging	Additional Results	Reference
αSyn/SNCA	mCherry-*αSyn* tg larvae	basal motility deficits and anxiety traits	-↑ brain apoptotic cells (acidine orange staining) -↓ TH^+^ in the telencephalon and PVZ at 5 dpf -Presence of pSer129-αSyn immunopositive inclusions at 5 dpf -αSyn-positive signal co-localized with Thioflavin S in several brain areas enriched in mCherry-αSyn expression	<RT-qPCR> -↓: *Syn1*, *Syn2a*, *Syn2b* and *Syn3* at 3dpf <Western blot> -↑: Syn IIb and Syn III at 5 dpf -Presence of high molecular weight pSer129-αSyn positive bands (phosphorylated mCherry-αSyn multimers)	[102]
	Dendra- *αSyn*-wt and A53T larvae	Normal escape responses to the tail touch stimulus	-↑ cell death TUNEL -Positive Thioflavin-S staining -Significantly smaller mitochondria, ↓ mitochondrial transport, and less total displacement of mobile mitochondria in Dendra-αSyn-A53T (NeuroD:mitoRFPTag) -Dendra-αSyn clearance kinetics in vivo: changes in the clearance rate of Dendra-αSyn-A53T after inhibition of autophagic flux by ammonium chloride	<Western blot> -αSyn: equivalent level in Dendra-αSyn-wt and A53T -pS129 αSyn: significant ↑ in Dendra-αSyn-A53T compared to αSyn-wt <Morphology> -Dendra-αSyn: significant ↓ length, bent spine, and smaller heads	[104]
PARKIN	MO-injected *parkin* KD larvae	No significant difference among wt, control MO, and Parkin-MO injected larvae	-Significant ↓ number of DC DA neurons by 20% -Significant ↓ by 50% following treatment of MPP^+^ -Electron microscopy: no definite morphological abnormalities of the mitochondria in parkin KD, but electron-dense material identified in the t-tubules	<Mitochondria respiratory chain function assay> -↓ complex I activity by 45%	[113]
	Antisense gripNAs mediated *parkin* KD larvae		-No loss of DC DA neurons -No alteration in mitochondrial morphology (mito-GFP) -Significant ↑ basal cell death (acridine orange) -↑ cell death in response to thermal stress (Heat shock)	<RT-qPCR> -No effect on *TH* mRNA levels <MMP> -No alteration (JC-1)	[154]
PINK1	MO-injected *pink1* KD embryos and larvae		-↑acridine orange accumulation in the brain and throughout the body at 24 hpf -Modest ↓ TH^+^ cells in the brains of 2 dpf by 30% -↓ expression of *reelin*, *Parkin* -↑ *fezl* and *Neurogenin-1* in the posterior DC -↓ slet-1 and acetylated tubulin staining in the spinal cord, suggesting loss of peripheral neurons	<Western blot> -↓: Serine 9-phosphorylated forms of GSK3 and active-catenin <Caspase 3-activity> -↑ Caspase-3 activity in MO-injected fish at 24 hpf. <MMP> -↓ (JC-1) <ROS> -Accumulation of the CM-H_2_DCFDA dye, in live 24 hpf <Morphology> -Long-tail, short-tail, and no-tail phenotypes (24 hpf) -Enlarged brain ventricle, slim tail-yolk extension, and curved spines (48 hpf)	[128]
	MO-injected *pink1* KD embryos and larvae	-Impaired response to tactile stimuli -↓ swimming behavior (↓ in swimming distance and speed)	-No significant alterations in the number of DA neurons in the vDC -Altered DA neuron patterning in the vDC: located more scattered and asymmetrically further from the midline, and shortened axonal projections, or had no projections to the forebrain	<Survival> -Fewer than 20% of the morphants survived past 10 days	[129]
DJ-1	MO-injected *dj-1* KD embryos and larvae		-No difference in the number of DA neurons -Significant ↓ DA neurons after exposure to H_2_O_2,_ compared with WT -Significant ↓ DA neurons after exposure to proteasome inhibitor MG132, compared with WT -Marked ↑ neuronal cell death after DJ-1inactivation and MG132 exposure (TUNEL)	<RT-qPCR> -↑: *SOD1* with and without exposure to H_2_O_2,_ *Catalase* only with exposure to H_2_O_2,_ *p53* and *Bax* with and without exposure to MG132	[136]
	*dj-1* KO adult			<Western blot> -↓: TH in the KO brains at the late adult stage <Proteomics> -Using the young adult (3-month) brain -Dysregulated proteins involved in mitochondrial metabolism, mitophagy, stress response, redox regulation, and inflammation influenced by the lack of *dj-1* <Morphology> -Lower body mass especially prevalent among male fish <In Gel Complex I Activity Assay> -↓ mitochondrial complex I activity over time	[138]
LRRK2	MO-injected *lrrk2* KD embryos and larvae		-Significant neuronal loss, including ↓ DA neurons -β-synuclein aggregation in the DC, midbrain, hindbrain, and postoptic commissure -Mis-localization of Na^+^/K^+^ ATPase protein in the apical and lateral side of the pronephric duct epithelial cells (basolateral in wt embryos)	<Western blot> -↑: β-synuclein, PARK13, and SOD1 <Morphology > -Developmental perturbations such as axis curvature defects, ocular abnormalities, and edema in the eyes, lens, and otic vesicles	[151]
	MO-injected *lrrk2* -WD40 deletion KD embryos and larvae	↓ swimming distance	-Loss of TH^+^/DAT^+^ DA neurons in the DC -↑ apoptosis (TUNEL) -↓ and disorganization of axon tracts, most prominently in the optic tectum (stained axonal microtubules using an acetylated-tubulin antibody)	<RT-qPCR> -↓: *TH* <Western blot> -↓: TH <Morphology> -Normal embryonic development, at least up to 7 dpf, without any distinguishable morphological defects, compared with *LRRK* KD showing embryonic lethality and severe developmental defects	[152]

↑, increase/upregulation; ↓, decrease/downregulation; αSyn/SNCA, α-synuclein; PARK2, PD Protein 2 Gene; PINK1, PTEN-induced putative kinase 1; PTEN, phosphatase and tensin homolog; PARK7, PD Protein 7 Gene; LRRK2, Leucine-rich repeat kinase 2; tg, transgenic; PVZ, periventricular zone; TH, tyrosine hydroxylase; wt, wild type; MO, morpholino oligonucleotides; KD, knockdown; KO, knockout; NA, nucleic acid; (v)DC (ventral)diencephalon; DA, dopaminergic; MMP, mitochondrial membrane potential; dpf, day post-fertilization; hpf, hour post-fertilization; ROS, reactive oxygen species; TUNEL, terminal deoxynucleotidyl transferase dUTP nick-end labeling; GSK, glycogen synthase kinase.

## 4. Investigation

### 4.1. Locomotor-Behavior Analysis

Locomotor activity in zebrafish larvae is evaluated as a functional indicator of potential neurobehavioral changes caused by chemical toxins or genetic modifications under alternating light and dark conditions [155]. Behavioral responses in zebrafish, including alterations in movement and locomotion patterns, are influenced by intricate neural circuits that encompass perception, cognition, decision-making, and visuomotor functions [27,155]. These responses can be diminished or intensified by various stimuli and have been investigated through a range of tests and devices, specifically in zebrafish [26]. To evaluate the effects of chemical exposure and genetic modification on larval motor function, larvae are positioned in a well plate, and automated video-tracking systems such as EthoVision (Noldus) are employed to analyze the total distance traveled, mean velocity, movement patterns, distance per bolt, bolt interval, and stimulus-evoked motor responses [156,157]. Zebrafish larvae exhibit strong and measurable locomotor reactions to sensory stimuli such as light flashes, which are influenced by dopamine signaling. This facilitates the identification of nuanced sensorimotor impairments, rendering zebrafish larvae exceptionally appropriate for investigating both the pathophysiology and functional ramifications of DA dysfunction during phases when therapeutic approaches may remain beneficial [26,158]. Simple locomotor and stimulus-evoked behavioral assays are generally feasible beginning at approximately 5–7 dpf, when zebrafish larvae develop sufficiently robust swimming and sensorimotor responses for reliable quantitative analysis [159,160,161]. In addition to automated larval swimming tracking, open-field locomotor assays can be used to assess the locomotor ability and turning angles of individual adult fish [162]. In an adult zebrafish PD model, Ashok et al. applied a stimulus-evoked C-bend behavioral paradigm to examine alterations in motor coordination and rigidity-associated responses [163]. By repeatedly delivering mechanical vibrational stimuli and monitoring escape-related body flexion behavior, the study identified marked declines in C-bend responsiveness after MPTP and rotenone treatment, suggesting progressive impairment of sensorimotor performance [163,164]. Adult zebrafish exhibit mature neural circuitry and more complex behavioral repertoires, allowing evaluation of anxiety-like behavior, social interaction, aggression, learning, and memory using established behavioral paradigms such as the novel tank test, shoaling assay, and social preference test [165,166,167,168]. Zebrafish exhibit well-documented responses of fear and anxiety, and they possess the capacity to learn complicated relationships [169]. Thus, non-motor phenotypes, such as spatial memory assessed via the Y-maze assay, anxiety levels measured through thigmotaxis, and social interactions, can be examined [30]. A recent study has further expanded zebrafish neurobehavioral assessments to include sleep- and sensory-related phenotypes relevant to prodromal PD symptoms [139]. Sleep-associated abnormalities, including altered sleep latency, daytime sleep ratio, and circadian locomotor activity [170], can be quantified using infrared beam-crossing–based Locomotor Activity Monitor systems during light/dark cycle recording [170,171]. In addition, sensory dysfunction can be evaluated using touch-evoked response assays, in which tactile stimulation is applied to the head or tail region, and immediate escape swimming responses are quantified [139]. These approaches enable the investigation of non-motor manifestations associated with early DA dysfunction in zebrafish PD models [170,172,173]. Furthermore, zebrafish are visually adept creatures capable of distinguishing colors and patterns and can establish associations through olfactory or auditory inputs [174,175]. Unlike certain murine motor behavioral paradigms that require repeated pre-training sessions, zebrafish locomotor assays generally do not require formal motor training prior to analysis [155]. However, short acclimation or habituation periods are commonly included before behavioral recording to reduce stress-related variability and stabilize swimming activity [30,156]. This modified behavior can facilitate the screening and identification of potential disease-modifying treatments during the pre-clinical development phase and can be clinically applied to neurodegenerative disorders, including PD.

### 4.2. Imaging

A transgenic line in which specific DA neurons are fluorescently labeled would be beneficial for investigating the development and degeneration of DA neurons in PD. Xi et al. developed a transgenic zebrafish line, Tg(dat:EGFP), in which green fluorescent protein (GFP) was expressed under the regulation of cis-regulatory regions of the DAT (*dat*) gene, in which DA neurons were labeled with GFP [176]. The authors performed whole-mount in situ hybridization (ISH) of th and *dat*, and double immunostaining for GFP with anti-GFP antibody (Ab) and TH with anti-TH Ab on forebrain cryosections [176]. Tg (flk1:GFP) zebrafish were used to evaluate changes in the neural vasculature [52,177,178,179]. For example, Wang et al. observed significant deterioration of the neurovascular system following MPTP treatment compared to that in the control group [177]. Co-treatment with *Calendula officinalis* L. extract (ECoL) and MPTP significantly mitigated the loss and disorganization of neural vasculature in the zebrafish brain induced by MPTP, suggesting a protective role for ECoL in the neural vasculature [177]. ISH is performed to localize the mRNA of a specific gene in the zebrafish brain using a gene probe. Cells that express a certain gene are identified using double-labeling with a marker protein for DA neurons (anti-TH) in a PD model, referred to as double-labeling fluorescence ISH with immunochemistry [180]. In addition to conventional static in vivo fluorescence imaging, recent advances have enabled real-time monitoring of pathological protein turnover in zebrafish PD models [104]. Son et al. [181] employed a photoconvertible Dendra-αSyn_A53T to longitudinally track αSyn degradation dynamics within living neurons. Using this live-imaging platform, the study demonstrated that ATP-citrate lyase inhibition enhanced in vivo αSyn clearance, underscoring the value of zebrafish models for dynamic evaluation of protein aggregation and autophagy-related processes [181].

ROS levels in zebrafish larvae were detected using a ROS assay kit and a fluorescent microscope, as described previously [52,157,182]. The accumulation of ROS, evaluated using the oxidized form of the cell-permeant ROS indicator acetyl ester of 5-(and 6-)chloromethyl-2,7-dichlorodihydrofluorescein diacetate (CM-H_2_DCFDA), can also be observed in living zebrafish [128] or ex vivo fluorometric biochemical ROS quantification [182]. Anichtchik et al. found that *pink1* morphants exhibited elevated ROS levels, as demonstrated by increased accumulation of the oxidized CM-H_2_DCFDA probe in 24 h post-fertilization (hpf) MO-injected zebrafish embryos, whereas treatment with 50 mM LiCl markedly reduced ROS overproduction [128]. To examine synaptic contact at the neuromuscular junction (NMJ), analysis of immunostained sections for the presynaptic marker synaptic vesicle protein 2 (SV2) and the postsynaptic marker α-bungarotoxin (α-BTX) is employed [183]. Vorhees et al. investigated the effects of 6-OHDA on olfactory synaptic connections. They identified a pattern of synaptic disorganization followed by recovery across all glomeruli, indicating the extensive influence of 6-OHDA injections throughout the olfactory bulb, as evidenced by sections immunostained against the presynaptic marker SV2, which labels the olfactory axon terminals [61]. The mitoGFP Tg(Hsa.Cox8a:MLSEGFP)^ia301^ line was used to evaluate mitochondrial mass and morphology by confocal microscopy, with subsequent signal quantification relative to wild-type controls [184]. The mitochondrial membrane potential (MMP) becomes depolarized during apoptosis [185,186]. MMP was assessed by quantifying the accumulation of the fluorescent potentiometric probe tetramethylrhodamine methyl ester (TMRM) in embryos at 72 hpf [187]. Embryos were treated with either a vehicle or 1 μM TR001 and subsequently incubated in the dark for 24 h with 300 nM TMRM and 1.6 μM cyclosporin H to inhibit the multidrug resistance pump. Following a quick rinse in fish water, 72 hpf embryos were embedded in 0.8% low-melting agarose and prepared for confocal microscopy [187]. Other studies have investigated MMP using JC-1 dye [188,189]. In normal MMP, JC-1 aggregates and exhibits red fluorescence, whereas depolarized MMP displays green fluorescence, indicating apoptotic cells [190]. The researchers detected less JC-1 aggregation in mitochondria isolated from *pink1* KO zebrafish, indicating a lower MMP [188]. More recently, Carneiro et al. evaluated MMP in a 6-OHDA-induced larval zebrafish PD model using a JC-1 fluorescent probe assay and demonstrated mitochondrial depolarization accompanied by increased apoptotic activity, supporting the involvement of mitochondrial dysfunction in DA neurotoxicity [189]. Quantification of apoptosis in zebrafish embryos is typically conducted using the dye acridine orange, which penetrates dead cells to bind to chromatin, followed by meticulous counting of stained cells under a microscope [191]. Acridine orange staining was performed according to the previously described standard protocol [102] to assess the extent of cell death. The researchers observed a notable increase in acridine orange-positive cells in the dorsal forebrain of F2 mCherry-αSyn tg embryos, particularly in the telencephalon and periventricular zone, in comparison to that in the control group [102]. A different method for detecting apoptosis is a terminal deoxynucleotidyl transferase dUTP nick-end labeling (TUNEL) assay. At 1 dpi of 6-OHDA, the authors noted a substantial increase in TUNEL+ cells within the glomerular layer of the olfactory bulb. At 3 dpi, the number of TUNEL+ cells reverted to control values, suggesting that apoptosis occurred swiftly and was not prolonged [61].

### 4.3. Gene Expression

Researchers have investigated the effects of toxins and genetic modifications on the expression of genes involved in neurodevelopment, oxidative stress, mitophagy, and neuroinflammation in a zebrafish PD model using RNA extraction and Reverse Transcription-quantitative Polymerase Chain Reaction (RT-qPCR) [29,52,192]. Furthermore, alterations in gene expression following the administration of the candidate drugs were validated using RT-qPCR [127,193,194]. Genes related to the hallmark of PD (*αSyn*, *th1*, *th2*, *dat*), neurodevelopment (*hoxb1a*,*tuba1b*, and *syn2α*), oxidative stress (*sod1*, *sod2*, *gss*, *gsto2*, *gpx4a*, and *cat*), pink1/parkin-dependent mitophagy (*pink1*, *parkin*, *fis1*, *atg7*, *atg12*, *ulk1b*, *beclin1*, *ambra1a*, and *lc3b*), the mTOR/FoxO signaling pathway (*foxO3a*, *mtor*, *ppargc1α*, *tsc1*, *prkaα1*, and *sesn2*) [52] and High-mobility group box 1 (HMGB1)-mediated inflammation (*hmgb1*, *tlr4*, *and nfkb*) [192] are the subjects of investigation. Using RT-qPCR, Kalyn et al. revealed that MPTP-treated larvae displayed 60% and 31% reductions in *th1* and *dat* expression, respectively, whereas *p53* expression increased by 35% compared to non-treated controls [29]. MPP+ exposure reduced *th1* and *dat* expression by 36% and 55%, respectively, while increasing *p53* expression by 39% compared with control fish [29]. Priyadarshini et al. demonstrated that oxidative stress (exposure to H_2_O_2_) markedly downregulates *th2* and upregulates *pink1* in larval zebrafish. Both effects were normalized by treatment with the drug L-glutathione reduced (LGR) [127]. LGR, in conjunction with *pink1* mRNA, could also restore both *th1* and *th2* cell populations that were diminished in *pink1* morphants [127]. Their following study validated the genes identified as altered in the microarray analysis using RT-qPCR, confirming the up-regulation of the *fibroblast growth factor receptor*, *fech*, *pax2a*, and *notch1a* genes and the down-regulation of the *sod3*, *hif1α*, *catalase*, and *atp1a2a* genes, which are important for hypoxia-induced factor signaling pathway in pink1 morphants [193]. Furthermore, they verified that all anomalies in gene expression patterns were rectified by mRNA injections, indicating that the modifications were mediated by *pink1* and not by off-target effects of the MO [193]. Bretaud et al. demonstrated that MO KD of *dj-1* alone led to elevated levels of *p53* and *Bax* expression prior to toxin treatment without significant neuronal cell death, indicating the subthreshold activation of cell death pathways due to dj-1 deficiency [136]. Inhibition of the proteasome results in augmented expression of *p53* and *bax*, accompanied by extensive neuronal cell death, suggesting a role for *p53* in neuronal cell loss associated with *dj-1* deficiency [136]. Zhang et al. [194] demonstrated that idebenone administration in an MPTP-induced zebrafish PD model restored the transcriptional levels of antioxidant-associated genes, including *nrf2* and *ho-1*, based on RT-qPCR analyses, indicating potential involvement of the Nrf2/HO-1 signaling pathway in reducing oxidative stress [195]. In the same study, RT-qPCR analyses further revealed increased expression of *pi3k*, *akt1*, and *akt2* following idebenone treatment [195], suggesting modulation of the PI3K/AKT signaling pathway associated with anti-apoptotic and neuroprotective effects [196,197].

### 4.4. Western Blot and Enzyme-Linked Immunosorbent Assay (ELISA) Analysis

The expression of proteins modified by genetic alterations and pharmacological administration associated with PD was evaluated using Western blot and enzyme-linked immunosorbent assay (ELISA) [19,25,45]. Western blot is particularly useful for investigating the time-dependent accumulation of misfolded proteins and clearance of αSyn [18,24]. In vitro investigation indicated that phosphorylation of Ser 129 facilitates the production of αSyn filaments and oligomers, implying that widespread phosphorylation of αSyn at Ser 129 in synucleinopathy-affected brains is a significantly pathological event [198,199]. Lopez et al. performed Western blot analysis for total αSyn and phosphorylated αSyn at position S129 (pS129) from 3 dpf zebrafish exhibiting pan-neuronal expression of Dendra-αSyn-wt and A53T, with tubulin levels serving as a loading control [104]. Phosphorylated αSyn levels were markedly elevated in fish harboring the A53T mutation compared with those expressing Dendra-αSyn-wt in the CNS at the same age, and although Dendra-αSyn protein expression was detectable from 1 dpf, phosphorylation at S129 was only observed at 3 dpf, indicating time-dependent αSyn phosphorylation [104]. Moriya et al. identified that unphosphorylated αSyn fibrils were released from macrophages and then taken up by neurons, while αSyn_A53T fibrils were accumulated within neurons [200]. Consequently, αSyn fibrils appear to originate from monomers inside macrophages and microglia, thereafter disseminating to neurons to promote αSyn aggregation, as demonstrated in transgenic zebrafish expressing the A53T mutant in macrophages and microglia using Western blot analysis [200]. Bicistronic or multicistronic expression vectors have been used in transgenic lines when the expression of multiple genes is required [201,202]. A self-cleaving porcine teschovirus-1 2A peptide (P2A), which replaced the internal ribosome entry site (IRES), demonstrated superior cleavage efficiency in zebrafish embryo lines as evidenced by Western blot analysis [202]. Western blot analysis showed that *pink1* MO injection induced a dose-dependent reduction in PINK1 expression in 24 hpf zebrafish, which was partially rescued by human *PINK1* mRNA overexpression, while *pink1* morphants exhibited decreased inactive Ser9-phosphorylated GSK3 and active β-catenin levels, indicating dysregulation of GSK3/β-catenin signaling [128]. A recent study by Wang et al. [179] demonstrated that cyflumetofen exposure significantly reduced the levels of tight junction-associated proteins, including ZO-1, Claudin-5, and Occludin, in zebrafish larvae, as determined by ELISA analysis. Since these tight junction proteins are essential for maintaining BBB permeability and endothelial barrier integrity, their downregulation suggests BBB disruption and increased vascular permeability following cyflumetofen exposure [203,204].

### 4.5. Proteomics

**Figure 1:**
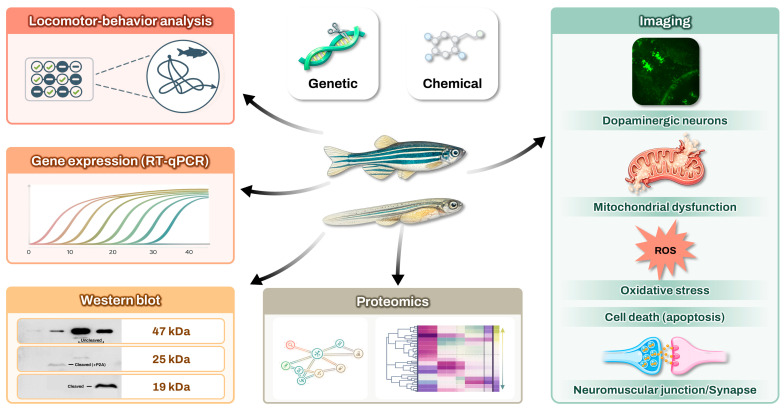
The investigative approach utilizing zebrafish models to elucidate the pathogenesis of Parkinson’s Disease and identify disease-modifying treatment.

Proteomic workflows used in zebrafish PD models have been described previously [53,205]. Proteomic analyses have revealed that neurotoxin-induced neurodegeneration in zebrafish is associated with alterations in pathways related to mitochondrial translation, vesicle trafficking, trans-synaptic signaling, oxidative stress, and proteasome-mediated protein catabolism [205,206]. Mitochondrial dysfunction induces excessive ROS and calcium imbalance, ultimately resulting in cellular apoptosis [5]. Maleski et al. reported coordinated dysregulation of DJ-1 (PARK7) in the protein–protein interaction network of MPP^+^-exposed larvae [53], suggesting activation of endogenous antioxidant defenses that are insufficient to overcome sustained mitochondrial dysfunction [207]. Lastly, the investigators observed alterations in protein degradation pathways, specifically the ubiquitin–proteasome system (UPS), with some proteasome subunits being significantly upregulated [138]. Elevated production of UPS-related proteasomes activates the UPS to counteract the buildup of misfolded proteins, such as αSyn, resulting from mitochondrial impairment and consequent ROS activation; nevertheless, sustained damage will ultimately undermine the UPS [208,209]. Researchers have identified 4091 proteins from the brains of young adult zebrafish, of which fewer than 5% exhibit altered expression levels in the *dj-1*/*park7* KO zebrafish model. These novel DJ-1-regulated proteins are associated with mitochondrial function, mitophagy, the stress response, and inflammation [138]. The investigative strategy employing zebrafish models to elucidate the pathophysiology of PD and identify disease-modifying treatment is summarized in Figure 1.

## 5. Future Direction

**Figure 2:**
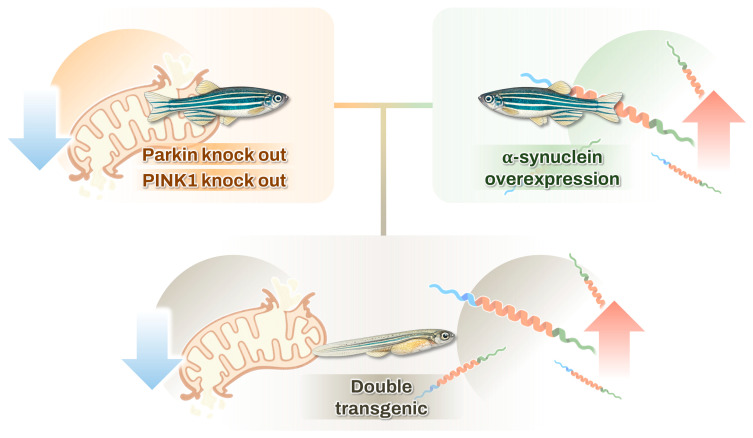
A double transgenic model created by mating an overexpression model with a gene knockout model to demonstrate the Parkinson’s disease phenotype during the larval stage.

Most genetic zebrafish PD models discussed in this review have been developed using MOs. Although MO-mediated gene KD is useful for rapid and transient functional analyses during early embryogenesis, several concerns remain regarding off-target effects, transient phenotypes, and the extent to which these models can faithfully recapitulate the progressive and chronic nature of PD [34,35,210]. To thoroughly investigate the pathogenesis of PD and conduct pharmacological screening for disease-modifying treatments using zebrafish models, it is essential to establish stable transgenic and KO models using targeted genetic editing techniques, such as CRISPR/Cas9, of genes associated with the condition [36,37,38]. It is essential to establish a reliable zebrafish model of PD that yields consistent behavioral and neurological outcomes. Because many zebrafish PD studies are conducted during the larval stage, the early manifestation of neurodegenerative phenotypes is particularly important for high-throughput drug screening. Owing to the progressive and self-amplifying nature of neurodegenerative diseases, future studies may benefit from the establishment of double transgenic (Figure 2) strategies combining αSyn overexpression with PD-associated gene KO models to accelerate pathological phenotypes during early development. Such approaches may facilitate earlier manifestation of DA neurodegeneration, mitochondrial dysfunction, and behavioral abnormalities in larval zebrafish, thereby improving the feasibility of large-scale drug screening. However, despite these potential advantages, double transgenic models may also increase developmental variability, genetic compensation, and early lethality, potentially complicating phenotype interpretation, reproducibility, and translational applicability [169,211]. Furthermore, excessive or artificial acceleration of pathological phenotypes may incompletely reflect the gradual progression of human PD. The double transgenic zebrafish PD models also still incompletely reproduce several hallmarks of human PD, including chronic progressive neurodegeneration and late-onset disease phenotypes, highlighting important translational challenges that remain unresolved [1,98].

Many of the investigations reviewed in the current study were conducted on larvae. The zebrafish lifespan is adequately short to minimize the duration of experiments, especially when the design necessitates the investigation of neurodegenerative processes, whereas it is sufficiently long to allow for the assessment of age-related aspects. A notable feature of zebrafish that is relevant to gene-based models is the substantial extent of post-embryonic neurogenesis that persists into adulthood [18]. In addition to larval studies, rearing larvae to adulthood for the examination of adult PD models in a disease-like state is also beneficial for investigating the cognitive decline and neurobehavioral impairments associated with PD, as adult zebrafish exhibit more sophisticated and complex neurobehavior, including cognition, anxiety-like behavior, social impairment, and learning and memory, than do larvae [30,31].

As previously mentioned, the regenerative capabilities of zebrafish have introduced certain challenges in PD investigations, complicating the construction of a zebrafish PD model. These regenerative properties may partially compensate for neuronal loss and thereby limit the faithful recapitulation of chronic neurodegeneration observed in human PD [62,63,64]. Consequently, caution is required when interpreting neurodegenerative phenotypes and therapeutic outcomes in zebrafish PD models. However, despite these limitations, understanding the endogenous regenerative mechanisms of zebrafish may provide valuable insights into neuronal repair pathways and facilitate the identification of novel regenerative therapeutic strategies applicable to human neurodegenerative disorders.

Emerging technologies are expected to substantially improve the precision and translational relevance of zebrafish PD models. In particular, single-cell RNA sequencing, spatial transcriptomics, proteomics, and multi-omics approaches may facilitate cell-type-specific characterization of DA neuronal vulnerability, neuroinflammatory responses, glial interactions, and molecular alterations during PD progression [212,213,214,215,216]. Furthermore, advanced in vivo imaging approaches enabled by zebrafish optical transparency, including calcium imaging and whole-brain live imaging, may allow real-time investigation of neuronal activity, αSyn propagation, mitochondrial dysfunction, mitophagy impairment, and neurodegenerative processes in living organisms [217,218]. The integration of these technologies with AI (artificial intelligence)-assisted behavioral tracking systems may further improve the sensitivity, reproducibility, and translational applicability of zebrafish-based PD research [219].

## 6. Conclusions

Zebrafish have emerged as valuable vertebrate models for investigating the pathophysiology of PD and for high-throughput screening of potential disease-modifying treatments. Chemical and genetic zebrafish PD models successfully recapitulate multiple pathological features of PD. In addition, the optical transparency, rapid development, and genetic manipulability of zebrafish provide substantial advantages for real-time in vivo imaging, phenotypic screening, and mechanistic investigation. Importantly, this review not only summarizes currently available zebrafish PD models but also integrates the diverse investigative methodologies used to evaluate these models. By systematically organizing both experimental strategies and representative findings, this review provides a practical framework that may assist researchers in designing, optimizing, and interpreting zebrafish PD studies for mechanistic investigation and therapeutic discovery. Nevertheless, several limitations remain. Many currently available zebrafish PD models rely on transient MO-based approaches and incompletely reproduce the chronic and progressive neurodegeneration observed in human PD. In addition, the remarkable regenerative capacity of zebrafish may complicate the interpretation of long-term neurodegenerative phenotypes. Therefore, future studies should focus on the development of stable transgenic and CRISPR/Cas9-based models with improved reproducibility and translational relevance. Furthermore, emerging technologies, including single-cell transcriptomics, integrated omics approaches, advanced live imaging, and AI-assisted behavioral analyses, are expected to further improve the precision and translational applicability of zebrafish PD research. Collectively, continued refinement of zebrafish PD models and investigative methodologies may facilitate a deeper understanding of PD pathogenesis and accelerate the identification of novel disease-modifying therapeutic strategies.

## Figures and Tables

**Table 1:** Comparative overview of chemical and genetic zebrafish models of Parkinson’s disease.

Model Category	Representative Models	Major Strengths	Major Limitations
Chemical models	MPTP/MPP^+^ 6-OHDA Rotenone Paraquat	rapid induction of PD-like phenotypes; relatively simple experimental procedures (e.g., immersion); facilitating high-throughput drug screening	Acute toxin-induced injury; systemic toxicity; developmental abnormalities; dose sensitivity; route-dependent variability; limited recapitulation of progressive neurodegeneration
Genetic models	SNCA Parkin/PARK2 PINK1 DJ-1/PARK7 LRRK2	Familial PD-associated pathway relevance; stable transgenic and CRISPR/Cas9-based knockout models; long-term molecular alterations and protein homeostasis; mechanistic investigation of PD-associated pathways	Transient suppression in MO-based knockdown models (e.g., off-target effects and variable phenotypes); time-consuming generation and maintenance of stable lines; genetic compensation; incomplete recapitulation of late-onset human PD

MPTP, 1-Methyl-4-Phenyl-1,2,3,6-Tetrahydropyridine; MPP^+^, 1-methyl-4-phenylpyridinium; 6-OHDA, 6-Hydroxydopamine; PD, Parkinson’s disease; SNCA, α-synuclein; PARK2, PD Protein 2 Gene; PINK1, PTEN-induced putative kinase 1; PTEN, phosphatase and tensin homolog; PARK7, PD Protein 7 Gene; LRRK2, Leucine-rich repeat kinase 2; CRISPR/Cas9, clustered regularly interspaced short palindromic repeats-associated protein 9; MO, morpholino oligonucleotides.

## Data Availability

No new data were created or analyzed in this study. Data sharing is not applicable to this article.

## Abbreviations

The following abbreviations are used in this manuscript:

PD	Parkinson’s disease
αSyn	α-synuclein
DA	dopaminergic
MO	morpholino oligonucleotides
KD	knockdown
KO	knockout
CRISPR/Cas9	clustered regularly interspaced short palindromic repeats-associate protein 9
MPTP	1-methyl-4-phenyl-1,2,3,6-tetrahydropyridine
MPP^+^	1-methyl-4-phenylpyridinium
DAT	dopamine transporter
BBB	blood–brain barrier
dpf	day post-fertilization
hpf	hour post-fertilization
dpi	day post-injection
6-OHDA	6-Hydroxydopamine
ROS	reactive oxygen species
MAO	monoamine oxidase
CNS	central nervous system
PQ	paraquat
LB	Lewy body
vDC	ventral diencephalon
SOD	superoxide dismutase
TH	tyrosine hydroxylase
Ab	antibody
GFP	green fluorescent protein
ISH	in situ hybridization
NMJ	neuromuscular junction
MMP	mitochondrial membrane potential
TMRM	tetramethylrhodamine methyl ester
TUNEL	terminal deoxynucleotidyl transferase dUTP nick-end labeling
RT-qPCR	reverse transcription-quantitative polymerase chain reaction
GSK	glycogen synthase kinase
UPS	ubiquitin–proteasome system
i.p.	intraperitoneal injection
MDA	malondialdehyde
GSH	glutathione
LGR	L-glutathione reduced
PTEN	phosphatase and tensin homolog
AR-JP	autosomal recessive juvenile Parkinsonism
AI	artificial intelligence
